# The Mediating Role of Academic Engagement in the Relationship Between Academic Hope and Academic Adjustment of Undergraduate Students: The Role of Gender as a Moderator

**DOI:** 10.1002/brb3.71079

**Published:** 2025-11-17

**Authors:** Kimia Mehrali, Maryam Mohsenpour, Abbas Abdollahi

**Affiliations:** ^1^ Department of Educational Psychology, Faculty of Education and Psychology Alzahra University Tehran Iran; ^2^ Department of Counseling, Faculty of Education and Psychology Alzahra University Tehran Iran

**Keywords:** academic adjustment, academic hope, engagement, gender, higher education, structural equation modeling

## Abstract

**Purpose:**

The process of academic adjustment is crucial for students to effectively adjust to the demands of higher education. This study aimed to examine the mediating role of academic engagement in the relationship between academic hope and adjustment among university students. Despite extensive research on academic hope, engagement, and adjustment, no prior studies have explored this mediating relationship.

**Methods:**

A total of 402 undergraduate students (200 women and 202 men) were selected using cluster sampling. Data were analyzed using structural equation modeling, and construct validity was evaluated through confirmatory factor analysis. Path analysis with bootstrapping was conducted to test the mediation model, and multi‐group analysis was used to assess gender as a moderating variable.

**Results:**

Findings from path analysis indicated that academic engagement significantly mediated the relationship between academic hope and academic adjustment. The proposed model accounted for 63% of the variance in academic adjustment. Furthermore, a multi‐group analysis revealed that the impact of academic hope on academic adjustment was stronger in men than in women.

**Conclusions:**

This study showed that academic hope, mediated by academic engagement, is associated with students' academic adjustment. The structural model confirmed that this relationship differs by gender, with notable differences between male and female students. The results of this study will contribute to the formulation of effective interventions and educational programs aimed at improving students' academic adjustment.

## Graphical abstract

Academic engagement significantly mediates the relationship between academic hope and academic adjustment among undergraduate students. The effect of academic hope on adjustment is stronger in men than women.

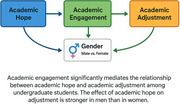

## 1 Introduction

University experience is an academically significant phase of students' lives, which usually goes along with the challenges of living outside their parents' home, building new social contacts, and facing academic pressures and financial stress (Duchesne et al. 2007). These challenges might inhibit adjustment to the new environment, and hence, academic adjustment becomes extremely important for academic achievement (Samari Safa and Poordel 2022).

According to Baker and Siryk (1986), academic adjustment refers to students' capability of handling obstacles and dealing with novel conditions in higher education. In short, this indicates the effectiveness of coping with academic pressure and striving for success (Li and Zhang 2022). According to self‐determination theory, motivation plays a core role in academic adjustment (Ryan and Deci 2000). Academic adjustment not only dictates students’ development and academic performance but also impacts their well‐being, self‐esteem, and future ambitions (Kiang and Fuligni 2010).

Given its importance, academic adjustment has been extensively researched in sociology, psychology, and education. Successful adjustment has been deemed an overriding determinant of success, which has encouraged researchers to examine the factors associated with it and its outcomes (Nasiri et al. 2017).

The study identified academic hope as the variable associated with students' academic adjustment. Snyder (1994) established the criterion of hope through his hope theory, initially developed within the framework of positive psychology. Snyder (2000) defined hope as a positive emotion that stems from an individual's assessment of their ability to achieve desired goals. Academic hope specifically refers to a person's belief or expectation regarding the attainment of favorable educational outcomes. It encompasses the perception that one has the necessary competencies to achieve academic goals and fulfill scholarly aspirations (Izadpanah and Rezaei 2022).

Academic hope, as formulated in positive psychology, is a multi‐component construct that has much in common with Snyder's hope theory. According to Snyder (2002), hope is a cognitive–motivational process involving two core components: agency thinking (having faith in one's power to initiate and persist in courses of action directed at goals) and pathway thinking (the ability to generate alternative paths to achieve goals). In the learning context, academic hope can be characterized as the practical implementation of hope theory, as students set valuable academic goals, plan alternative means of reaching them, and preserve the faith necessary to endeavor to reach these goals despite adversity. Empirical research supports this theoretical connection. So, for instance, Day et al. (2010) demonstrated that hope constitutes a unique and powerful prognosis of performance on college campuses, which exceeded in explanatory power the abilities of intelligence, personality, and prior performance.

The items included in the Academic Hope Questionnaire used in this study may, at first, appear to correspond with the construct of academic aspirations, in that they bear a tendency to address future‐oriented education outcomes such as securing a job, achieving economic security, or receiving social status. Academic aspiration defines students’ preferred and expected levels of education and occupational achievement and constitutes students’ long‐term goals (Khattab 2015). In contrast, academic hope, as proposed in Snyder's (2000) hope theory, is conceptualized as a cognitive–motivational process in the form of two intertwined components: agency thinking (the perceived capability to initiate and sustain movement in the direction of goals) and pathways thinking (the ability to generate multiple means of achieving those goals).

It is a key cognitive–motivational principle that can support students in shaping their attitudes and behaviors toward academic endeavors. Academic hope fosters a positive mindset among students, encouraging them to embrace the educational environment and engage in learning activities with increased efficiency. Enhancing levels of academic hope can lead to greater motivation and persistence, thereby improving academic performance and achievement (Esmaeili et al. 2019). Consequently, academic hope plays a critical role in educational settings, as it involves goal‐setting strategies for achieving those goals and reinforcing one's determination to pursue them (Feldman and Kubota 2015). In this context, hope functions as an active agent that enables individuals to adjust to unsatisfactory situations and provides them with the resilience needed to attain their objectives (Cheraghian et al. 2023).

One of the variables examined in this research is academic engagement. Previous studies, such as Wang et al. (2021) have demonstrated a strong correlation between academic adjustment and academic engagement. Academic engagement is associated with higher levels of academic adjustment, as engaged students tend to develop positive attitudes and motivation toward their coursework. This engagement leads to more active participation in class and a greater investment of effort in their studies. Consequently, students are more likely to develop effective adjustment strategies, such as time management and study skills that enhance their academic performance and their ability to adjust to university life. Engaged students are more motivated, develop effective study habits, and adjust more successfully to university life (Reschly and Christenson 2022).

Engagement also fosters belonging, collaboration, and meaningful interactions with peers and instructors, further supporting learning (Gasiewski et al. 2012). Despite extensive research on academic adjustment, the mediating role of academic engagement in the relationship between adjustment and hope remains underexplored (Marsh et al. 2011).

Accordingly, the present study seeks to examine how academic engagement mediates the relationship between academic hope and academic adjustment, addressing a current research gap among public university students in Tehran. This study aims to offer a novel contribution by focusing on cognitive objectives while considering emotional, motivational, and behavioral development, drawing upon existing research literature. The study examines the role of gender as a moderating variable in the relationships among these factors, acknowledging that previous research on gender has yielded conflicting results. The findings of this study have the potential to offer valuable insights for educators and students, contributing to the understanding of strategies that enhance educational success.

### 1.1 Direct Effect of Academic Hope and Indirect Effect of Academic Engagement

Michaeli Monee et al. (2015) conducted a study that demonstrated a significant positive correlation between academic hope and adjustment. Students with high levels of hope tend to be more energetic and proactive in setting and pursuing their goals, which enhances their adaptability within academic environments. Hope, as a component of psychological capital, serves as a valuable resource, facilitating students' academic adjustment by enabling them to navigate and thrive in educational settings (Hazan Liran and Miller 2019).

Based on the reviewed literature, we propose the following hypothesis for testing in this study, which could significantly advance our understanding of student academic adjustment:

H1: Academic hope directly and significantly affects academic adjustment.

Gil‐Doménech and Berbegal‐Mirabent (2019) showed that motivated students actively participate in their academic activities and learning processes. Increased motivation leads to better academic performance and a deeper understanding of the subjects. Such students are more likely to attend classes regularly, actively participate in discussions, complete their assignments on time, and seek additional learning opportunities. Moreover, they are more inclined to develop critical thinking skills, problem‐solving abilities, and effective study habits. This proactive approach creates optimal learning conditions, strengthens their sense of inclusion, and fosters rapport among students and between students and teachers, ultimately enhancing the educational process. These points make it clear that academic engagement is crucial in school and warrants careful consideration.

Studies have shown that academic engagement relates to adjustment (Wang et al. 2021). In support of this, Eghdami and Yousefi (2022) demonstrated that academic engagement influences students' adjustment to university. One effective strategy to support students' adjustment to college is to focus on enhancing their academic engagement. Academic hope can also be associated with engagement through various pathways. First, it may act as a motivational force, inspiring students to strive and excel in their academic work. Second, it can influence the goal‐setting process and the effort invested in achieving those goals. For instance, academically hopeful students are more likely to set ambitious goals and develop strategies to overcome obstacles.

More importantly, academic hope may influence students' emotional experiences by nurturing positive feelings, such as curiosity and enjoyment in learning. Low levels of academic hope can lead students to experience negative emotions, such as fear, which may reduce their level of engagement. Academic hope plays a significant role in motivating goal‐setting, goal‐directed behavior, and emotional experiences, all of which are integral to academic engagement (Azadianbojnordi et al. 2022). Despite the existing literature highlighting the critical impact of academic engagement on key outcome variables, such as academic adjustment and hope, there is a notable lack of research examining academic engagement's mediating role between academic hope and adjustment. Therefore, the hypotheses underpinning this study are as follows:

H2: Academic hope directly and significantly affects academic engagement.
H3: Academic engagement directly and significantly affects academic adjustment.
H4: Academic hope is associated with academic adjustment through the mediation of academic engagement.

### 1.2 Gender's Role as a Moderator

Akhtar and Alam (2016) represented one of the investigations focused on gender roles in academic adjustment. This particular study revealed differences in academic adjustment among male and female students. One of the findings from Singh et al. (2023) indicated that no significant differences exist between genders regarding academic adjustment, which challenges our understanding of this issue. Similarly, the research on academic hope has produced contradictory results. Griggs and Crawford (2019) argued that there is no evidence to support the belief that males and females differ in the extent of their hope. Ganji et al. (2021) suggested that male students report higher levels of academic hope compared to their female classmates. Dami et al. (2020) indicated that female students exhibit greater academic hope regarding future educational opportunities than their male counterparts. This paradoxical pattern is also evident in the significant influence of gender on academic engagement. In this regard, Tortosa Martínez et al. (2023) found no significant differences in the levels of academic engagement between males and females. However, other studies, Derrick et al. (2022) and Lawson and Salter (2023) identified significant effects of gender on academic engagement in various contexts, raising awareness of this issue. Individuals can observe these differences in their daily activities.

The conflicting research findings regarding the influence of gender on academic adjustment and engagement highlight the need to investigate the moderating role of gender in the relationship between academic adjustment and academic engagement, collectively referred to as academic hope. Thus, the following hypothesis is proposed:

H5: Gender plays a moderating role in the research model.

## 2 Methods

### 2.1 Sampling and Procedure

We gathered the data for this study using the Academic Adjustment Scale (Anderson et al. 2016), the Academic Hope Scale (Khormaei and Kamari 2021), and the Student Academic Engagement Scale (Alonso‐Tapia et al. 2023). The researcher visited the target universities, obtained permission from the relevant professors, and requested that they allocate 15 min of class time for data collection. After entering the classroom with physical copies of the questionnaires, the researcher introduced them, explained the research project, obtained students' consent to participate, and rigorously followed ethical considerations throughout the data collection process. This process lasted a total of 1 month.

The statistical population of this study included all undergraduate students enrolled in public universities in Tehran during the 2023–2024 academic year. To select a representative sample from this population, a multi‐stage cluster sampling method was employed. First, several public universities offering undergraduate programs were identified. In the first stage, universities were selected based on program diversity and accessibility. In the second stage, faculties within these universities were treated as clusters, and a random selection of faculties was made (e.g., engineering, basic sciences, humanities, and social sciences). In the final stage, students from the selected faculties were randomly invited to participate. Participants were required to be undergraduate students currently enrolled in any of the selected universities during the 2023–2024 academic year. Only students who provided informed consent and were present at the time of data collection were included. Students with incomplete questionnaires or who withdrew during the survey were excluded from the analysis. A total of 420 students were invited, of whom 402 completed the questionnaires, resulting in a response rate of 95.7%.

After analyzing the data, we determined the final sample size to be 353 students, with 179 women (50.7%) and 174 men (49.3%). This gender balance underscores the inclusivity of the study and ensures a representative perspective. We selected the participants from the faculties of engineering, basic sciences, humanities, and social sciences.

It should be noted that, structural equation modeling (SEM), particularly using the maximum likelihood estimation approach, is significantly sensitive to the effects of outliers, which may lead to incorrect results. Therefore, outlier removal is a crucial precondition for applying the maximum likelihood strategy in AMOS (Byrne 2016).

Before running the main analyses, the dataset was checked for multivariate normality and possible outliers. Multivariate outliers were determined based on Mahalanobis distance values (*D* ^2^) computed by AMOS. AMOS provides two probability values, *p*1 and *p*2, for each case, where *p*2 is a criterion for the significance level of Mahalanobis *D* ^2^ according to the chi‐square distribution. According to conventional recommendations for screening SEM data (Byrne 2016; Kline 2023), cases with *p*2< 0.10 were deemed potential multivariate outliers and excluded from further analysis. A relatively conservative criterion value of 0.10 was used here to provide maximal assurance that the retained data assumed multivariate normality. After using this criterion, 49 cases were excluded, leaving 353 across which final analysis was conducted.

Participants from the faculties of engineering and basic sciences totaled 170, accounting for 48.2% of the sample, while those from the faculties of humanities and social sciences numbered 183, making up 51.8%. Thus, the student population exhibited a near balance in gender representation.

### 2.2 Data Analysis

We scored the questionnaires after data collection, defined the variables in SPSS software (version 26), and then entered the data. A comprehensive analysis of descriptive statistics was performed, which included the calculation of the mean and standard deviation, identification of outliers, assessment of missing data, evaluation of the normality of data distribution, determination of minimum and maximum scores, and examination of correlations between variables.

We employed confirmatory factor analysis (CFA) to assess the validity of each scale's psychometric properties. We estimated the reliability of the scales using McDonald's omega and Cronbach's alpha. We compared the fit indices of each scale against acceptable thresholds to ensure the accuracy and robustness of the evaluation. We conducted SEM using AMOS software‐24 version to explore the mediating role of academic engagement in the relationship between academic hope and academic adjustment.

### 2.3 Measures

The assessment of academic adjustment was carried out using the scale developed by Anderson et al. (2016), which consists of nine items rated on a 5‐point Likert scale. The Persian version of this scale, which was culturally adapted and validated by Baharvand et al. (2020), was employed in the present study. During the adaptation process, all items were reviewed for linguistic and cultural equivalence, and a pilot test was conducted among Iranian university students to ensure clarity and comprehension of the items.

The components of this questionnaire include Academic Lifestyle (e.g., “I derive pleasure from the lifestyle of a student”), Academic Success (e.g., “I am satisfied with my learning abilities at university”), and Academic Motivation (e.g., “My purpose for studying is to attain an improved lifestyle”). McDonald's omega and Cronbach's alpha measured the reliability of this scale at 0.73, indicating a satisfactory level of internal consistency. CFA evaluated the construct validity, revealing that all items had factor loadings exceeding 0.25, ranging from 0.33 to 0.98. The Academic Success subscale demonstrated the highest factor loading at 0.98. The fit indices for academic adjustment (CMIN/DF = 2.36, IFI = 0.95, GFI = 0.97, AGFI = 0.99, CFI = 0.95, and RMSEA = 0.06) were all within acceptable thresholds, confirming the model's suitability.

The measurement of academic hope was conducted using a 27‐item scale rated on a 5‐point Likert scale (Khormaei and Kamari 2021). This scale was originally developed and validated in the Iranian cultural context by its authors; therefore, no additional translation or cultural adaptation was required for its use in the present study. This scale comprises four subscales: Hope for Gaining Opportunities (e.g., “I hope that studying will facilitate my search for a suitable job in the future”), Hope for Acquiring Life Skills (e.g., “I hope that the knowledge I gain from university and textbooks will equip me with essential life skills”), Hope for the Utility of University (e.g., “The knowledge acquired at university has no association with future job success”), and Hope for Achieving Competence (e.g., “By pursuing higher education, I can earn the admiration of others”).

This questionnaire was chosen because of the specific focus of the researchers on university students’ academic hope, in contrast to Snyder's Adult Hope Scale, which primarily concerns general life goals and one's perceived ability to attain them (Snyder 2002). The present questionnaire, however, has objectives of immediate nature concerning learning outcomes at university, that is, future occupation, social and vocational abilities, and professional career. The questionnaire, therefore, accordingly suits the examination of hope in the learning process and university context. Despite, however, in still keeping within the scope of the cognitive–motivational dimensions of hope, the tool accounts for the learning environment as well in order to offer a more specific examination of the combination of hope and university performance. In essence, questionnaire administration permits the measurement of students’ academic hope based on realistic and feasible learning goals without expanding the scope to general life goals of adults.

The reliability of the scale, evaluated using McDonald's omega (0.73) and Cronbach's alpha (0.73), indicated a satisfactory level of internal consistency. CFA assessed construct validity, with factor loadings ranging from 0.48 to 0.78. The subscale Hope for Gaining Opportunities demonstrated the highest factor loading at 0.94. However, to ensure validity, we removed the subscale Hope for the Utility of University from the questionnaire due to its negative factor loading. The fit indices for academic hope (CMIN/DF = 2.85, IFI = 0.91, GFI = 0.89, AGFI = 0.84, CFI = 0.91, and RMSEA = 0.07) were all within acceptable ranges, confirming the suitability of the model.

We conducted the assessment of academic engagement using the Student Academic Engagement Scale (Alonso‐Tapia et al. 2023). The Persian version of this scale, adapted and validated by Mohsenpour et al. (2024), was used in the current study. The adaptation process involved reviewing all items for cultural and linguistic equivalence and conducting pilot testing to ensure clarity and contextual appropriateness for Iranian students.

It consists of 40 items rated on a 5‐point Likert scale. Each set of 8 items focuses on different aspects of person–situation interaction. The scale includes four subscales: The agency subscale, for example, states, “I endeavor to establish connections between the text I am reading and my interests to enhance my comprehension of the material.” The behavioral subscale, for example, states, “If I find something difficult to understand, I usually abandon it and focus on something else or change my activity.” For example, the cognitive subscale states, “Frequently, I prioritize assessing my teammates' proposals for resolving a problem, formulating a project, and so on,” while the emotional subscale states, “While doing practical assignments, I feel good because they resemble challenges that I enjoy solving.”

McDonald's omega and Cronbach's alpha evaluated the scale's reliability and found it to be 0.83, indicating a satisfactory level of internal consistency. CFA checked the construct validity, revealing factor loadings ranging from 0.36 to 0.66. The Cognitive Engagement subscale had the highest factor loading at 0.97. The fit indices for academic engagement (CMIN/DF = 2.58, IFI = 0.85, GFI = 0.89, AGFI = 0.85, CFI = 0.85, and RMSEA = 0.06) were all within acceptable thresholds, indicating an appropriate fit for the model.

## 3 Results

### 3.1 Descriptive Statistics

The current study contained no missing data, as every questionnaire returned by the participants was meticulously examined to ensure that all items were addressed. Tables 1 and 2 present descriptive statistics for the entire sample, categorized by gender. Table 1 presents the maximum and minimum scores for each subscale, as well as the mean, standard deviation, skewness, and kurtosis, used to assess the normality of the data distribution. We determined the data distribution in this study to be normal based on the obtained values.

**TABLE 1:** Descriptive statistics (minimum, maximum, mean, standard deviation, skewness, and kurtosis).

Constructs	Indicators	Mean	SD	Skewness	Kurtosis	Min	Max
Academic adjustment	Academic Lifestyle	10.36	2.48	−0.26	−0.36	4	15
	Academic Success	10.77	2.58	−0.40	0.16	3	15
	Academic Motivation	7.61	1.91	−0.76	0.28	2	10
Academic hope	Hope for Gaining Opportunities	22.13	4.08	−0.58	0.57	7	28
	Hope for Acquiring Life Skills	22.64	4.60	−0.13	0.14	8	32
	Hope for Achieving Competence	11.95	2.50	−0.51	0.80	4	16
Academic engagement	Agency	29.36	3.52	0.05	0.14	19	40
	Behavioral	28.32	3.85	0.003	0.17	17	38
	Cognitive	28.17	3.65	−0.14	0.70	12	39
	Emotional	30.32	3.97	−0.25	0.30	17	40

**TABLE 2:** Descriptive statistics for gender groups.

Constructs	Indicators	Male				Female			
		Mean	SD	Min	Max	Mean	SD	Min	Max
Academic adjustment	Academic Lifestyle	10.66	2.16	4	15	10.34	2.39	4	15
	Academic Success	10.96	2.29	6	15	10.96	2.16	6	15
	Academic Motivation	8.16	1.47	3	10	7.59	1.68	4	10
Academic hope	Hope for Gaining Opportunities	22.88	3.50	13	28	21.77	3.64	14	28
	Hope for Acquiring Life Skills	23.82	3.90	15	32	22.50	4.11	13	32
	Hope for Achieving Competence	12.35	2	8	16	11.86	2.12	6	16
Academic engagement	Agency	29.69	3.27	21	40	29.26	3.08	22	37
	Behavioral	28.67	3.59	19	38	28.57	3.14	21	38
	Cognitive	28.63	3.35	21	39	28.72	3.14	21	37
	Emotional	31.04	3.71	23	40	30.75	3.13	24	38

The results presented in Table 2 indicate that men had higher average scores than women on the academic motivation indicator of academic adjustment. Men outperformed women on average in the construct of academic hope across all three subscales: Hope for Achieving Competence, Hope for Gaining Opportunities, and Hope for Acquiring Life skills. Within the academic engagement construct, men scored higher on average than women in the emotional indicator, while the other indicators exhibited nearly equal average scores.

### 3.2 Examination of the Conceptual Model of the Research

**FIGURE 1:**
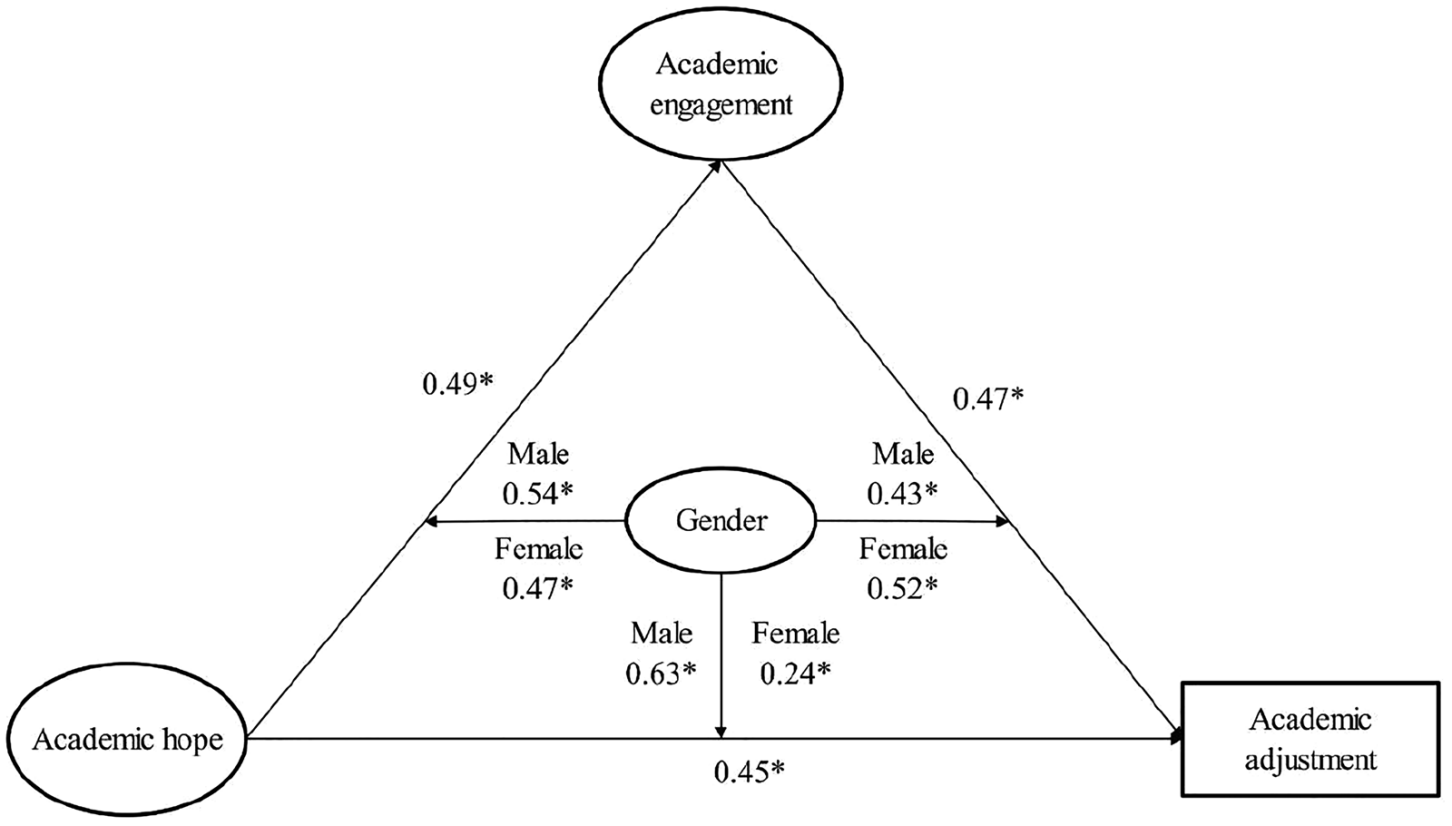
Summary of model results.

We used SEM to look at the conceptual model of the study. The goal of this study was to find out how academic engagement affects the relationship between academic hope and academic adjustment. Figure 1 illustrates the structural model of the study, displaying the standardized regression weights (*β*). We analyzed the model fit indices to evaluate the data's alignment with the designed model. The results indicated that the fit indices (CMIN/DF = 2.50, IFI = 0.96, GFI = 0.95, AGFI = 0.92, CFI = 0.96, and RMSEA = 0.07) were acceptable, signifying a satisfactory fit between the data and the research model. The proposed model explained 63% of the variance in academic adjustment, suggesting that the variables of academic hope and academic engagement accounted for 63% of this variance. Academic hope accounted for 48% of this variance, while academic engagement accounted for 15%.

### 3.3 Direct‐Effect Hypotheses

As shown in Figure 1 and Table 3, the examination of Hypothesis 1 through SEM tests revealed a significant direct relationship between academic hope and academic adjustment (*β* = 0.45, *p* < 0.05). Consequently, the confirmation of our first research hypothesis demonstrated substantial direct relationship between academic hope and academic adjustment. The results pertaining to Hypothesis 2 indicated a significant direct relationship between academic hope and academic engagement (*β* = 0.49, *p* < 0.05). Thus, the results also confirmed our second research hypothesis, indicating a notable direct relationship between academic hope and academic engagement. Hypothesis 3 posited that academic engagement would have a significant direct relationship with academic adjustment (*β* = 0.47, *p* < 0.05). Therefore, the confirmation of the third hypothesis demonstrated a significant direct relationship between academic engagement and academic adjustment.

**TABLE 3:** SEM results for academic adjustment, academic hope, and academic engagement.

Paths		*β*	*p*‐value
Direct paths	AH → AA	0.45	0.001*
	AH → AE	0.49	0.001*
	AE → AA	0.47	0.001*
Indirect paths	Mediation of AE	0.23	0.005*

Abbreviations: AA, academic adjustment; AE, academic engagement, AH, academic hope.

*N* = 353; *p < 0.05.

### 3.4 Indirect‐Effect Hypothesis

The bootstrap method was employed to investigate the fourth hypothesis, demonstrating that academic engagement serves as a significant mediator in the relationship between academic hope and academic adjustment (*β* = 0.23, *p* < 0.05). When academic engagement was incorporated into the analysis of the relationship between academic hope and academic adjustment, the strength of the relationship between these two variables decreased (from *β* = 0.45 to *β* = 0.23). We confirmed the fourth hypothesis, which suggests that academic hope can both directly and indirectly influence academic adjustment through the mediating effect of academic engagement. For a comprehensive understanding of these findings, please refer to Table 3.

### 3.5 Moderation Hypothesis

Hypothesis 5 examined the moderating role of gender in the direct and indirect paths of the research model through multi‐group analysis. Initially, we analyzed the standardized regression weights (*β*) and significance levels (*p*) for direct and indirect paths separately for men and women. We evaluated the significance levels for each path in the conceptual model to ascertain whether gender moderated the research model.

We assessed fit indices for female and male models. In the female model, the fit indices were as follows: CMIN/DF = 1.55, IFI = 0.97, GFI = 0.94, AGFI = 0.91, CFI = 0.97, and RMSEA = 0.05, all indicating an acceptable level of fit. Similarly, in the male model, the fit indices were CMIN/DF = 1.30, IFI = 0.98, GFI = 0.94, AGFI = 0.91, CFI = 0.98, and RMSEA = 0.04, which also reflected an acceptable level and remained nearly within the same range. The most critical fit indices, such as CMIN/DF, CFI, and RMSEA, were almost identical for models.

Table 4 shows that the model's direct paths had significant effects: from academic hope to academic adjustment (women: *β* = 0.24, *p* < 0.05; men: *β* = 0.63, *p* < 0.05); from academic hope to academic engagement (women: *β* = 0.47, *p* < 0.05; men: *β* = 0.54, *p* < 0.05); and from academic engagement to academic adjustment (women: *β* = 0.52, *p* < 0.05; men: *β* = 0.43, *p* < 0.05). Therefore, we identified a significant relationship between the research variables in female and male groups. Upon examining the standardized regression weights (*β*) in Figure 1, it is evident that differences exist between women and men in the *β* values for each path in the model. Consequently, we investigated the moderating role of gender to determine whether these differences between the male and female groups were significant.

**TABLE 4:** SEM results and multi‐group analysis for gender groups.

Gender	Male		Female		Multi‐group analysis
Paths	*β*	*p*‐value	*β*	*p*‐value	*p*‐value
AH → AA	0.63	0.001*	0.24	0.001*	0.007*
AH → AE	0.54	0.001*	0.47	0.001*	0.59*
AE → AA	0.43	0.001*	0.52	0.019*	0.47*

Abbreviations: AA, academic adjustment; AE, academic engagement; AH, academic hope.

*N* = 353; *p < 0.05.

As shown in Table 4, the analysis reveals that the path from academic hope to academic adjustment is statistically significant (*p* < 0.05). This finding suggests that gender modifies this relationship, implying that the impact of academic hope on academic adjustment varies between male and female groups. The analysis reveals that on the path from academic hope to academic engagement, the *p*‐value is greater than 0.05, suggesting that gender does not moderate this relationship. Thus, the effect of academic hope on academic engagement is consistent for men and women. Finally, when it comes to the path from academic engagement to academic adjustment, a *p*‐value of less than 0.05 suggests that gender does not play a moderating role in this relationship. This implies that the impact of academic engagement on academic adjustment is comparable for both male and female participants.

## 4 Discussion

This study investigated how academic engagement mediates the relationship between academic hope and academic adjustment among students from Tehran's public universities, while considering gender as a moderating factor. SEM supported the hypothesized associations, showing that academic hope is both directly and indirectly related to academic adjustment through engagement.

Consistent with prior research (Hazan Liran and Miller 2019; Michaeli Monee et al. 2015), the findings confirm that students with higher academic hope tend to adjust better to academic life. Unlike previous studies that focused on general psychological hope, the present research specifically examined academic hope as a domain‐specific construct and verified its influence on adjustment among a large and diverse group of undergraduates. Academic hope appears to serve as a motivational resource that fosters optimism, competence, and life skills—factors that facilitate effective adjustment and social integration within the university environment.

The second hypothesis, proposing a positive correlation between academic hope and engagement, was also supported and aligns with previous findings (Azadianbojnordi et al. 2022; Tomás et al. 2020; Vazirimehr and Salimi 2022). Extending this line of research to higher education, our results demonstrate that hopeful students are more likely to invest effort, persist in the face of obstacles, and engage meaningfully with academic material. This reinforces the view that academic hope promotes self‐regulated learning and fosters deeper educational engagement.

Similarly, the positive association between academic engagement and adjustment is consistent with earlier studies (Eghdami and Yousefi 2022; Wang et al. 2021). Our study contributes by conceptualizing adjustment as a multidimensional construct—encompassing academic lifestyle, success, and motivation—thus broadening the understanding of how engagement supports students’ adjustment. Engaged students are more likely to employ diverse learning strategies, participate actively in class discussions, and demonstrate flexibility in coping with academic demands.

The mediating role of academic engagement provides novel evidence regarding the mechanism through which academic hope enhances adjustment. Hopeful students tend to activate cognitive, emotional, and behavioral engagement processes, which in turn facilitate academic and social adjustment. This integrative pathway empirically validates prior theoretical assumptions (Azadianbojnordi et al. 2022; Eghdami and Yousefi 2022; Hazan Liran and Miller 2019; Michaeli Monee et al. 2015; Tomás et al. 2020; Vazirimehr and Salimi 2022; Wang et al. 2021). These findings extend the cross‐cultural validity of hope and engagement theories within non‐Western educational contexts. From a practical standpoint, interventions designed to strengthen students’ academic hope and engagement—such as motivational workshops, mentorship programs, and goal‐setting activities—may substantially improve students’ adjustment, persistence, and psychological well‐being.

In the factor analysis, the “Hope for the Utility of University” subscale had a negative loading and was thus excluded from the final model. While statistically merited, there are several conceptual explanations for this result. One is the possibility that some items did not entirely translate well into the cultural environment or the participants' daily realities, perhaps minimizing their applicability. In addition, the wording of some items may have caused ambiguity, which would have decreased their clarity and interpretability. It is possible that, in this population, the utility of university education is conceived in normative or symbolic terms rather than purely instrumental terms. Accordingly, the subscale may not have reflected the desired dimension, and its exclusion from the final model would be warranted both statistically and theoretically.

Nonetheless, questionnaire items, though largely concerning desired outcomes, implicitly measure the cognitive–motivational aspects of hope. Thus, items like “I hope that education will offer me opportunities to be successful in life” indicate not just aspirations but also goal‐striving expectations that lend energy to pursuing goals. There is empirical evidence to support the connection between academic hope and end goals, as well as students' beliefs concerning the worth and efficacy of accessible educational options (Day et al. 2010). Thus, the questionnaire assesses a mix of future aspirations and motivated beliefs regarding the usability of education and can be defended within Snyder's hope theory, as the expectation of attaining desirable outputs generates the mental energy needed to persist and exert effort (Gallagher et al. 2017).

Gender moderation analysis revealed that, while gender did not affect the associations between hope–engagement or engagement–adjustment, it did moderate the link between hope and adjustment. Academic hope had a stronger effect on adjustment among men than among women. Ryan and Deci's (2000) self‐determination theory illuminates this finding by positing that motivation significantly influences individuals' adjustment. Enhancing autonomy can bolster intrinsic motivation in a supportive environment, leading to improved adjustment. As shown in Table 2, average scores for the academic motivation component and each of the three components of academic hope were higher for men than for women. This theory‐based explanation suggests that men displayed higher motivation levels, facilitating greater adjustment and increasing hope within their educational settings.

In many cultural traditions, men, under the influence of internalized gender expectations maximizing self‐reliance, independence, and agency, often exhibit a powerful motivation for the achievement of personal and occupational aspirations. The concept of academic hope, and in particular the agency facet of hope, may facilitate increased intrinsic motivation and academic adjustment (Snyder et al. 2002). Moreover, from the perspective of expectancy–value theory, socio‐cultural influences such as gender roles, social beliefs, and teacher and parent expectations impact individuals’ success expectancies and task values; in males, this motivational system may deepen the agency of hope as a motivator for persistence in academic activity (Wang and Degol 2017; Wigfield and Eccles 2000). Once again, based on the evidence, hope and optimism are the most powerful antecedents of academic attainment and mental well‐being, and the phenomenon may have a stronger impact on males in achievement‐supporting social environments based on personal achievement (Marques et al. 2017).

The noted disparity in academic hope scores for men and women has causes in both socio‐cultural and methodological levels. Men tend to have greater academic hope by virtue of social demands and masculine roles in society in preparing for success in their profession and in providing for the family (Eagly and Wood 2016). Such demands are exerted by the family and the media and can have a bearing on academic hope (Chen et al. 2024). To eliminate such disparities, cultural perceptions must shift and the education system must ensure equal opportunities (Samoy et al. 2025). Again, the instruments of measurement may suffer from bias; scales focusing on professional aims may have the unintended effect of scoring higher in men (Cervone et al. 2006). As well, bias in the items of the questionnaire may distort the accuracy of measurement, in the event the questions have more pertinence in the life of one gender group (Steele and Aronson 1995).

In conclusion, this study contributes novel evidence from a non‐Western higher education context by integrating academic hope, engagement, and adjustment into a unified structural model and examining the moderating effect of gender. Theoretically, it advances understanding of the motivational and behavioral mechanisms that underlie academic adjustment. Practically, the findings suggest that fostering hope‐oriented engagement through targeted interventions could strengthen students’ motivation, resilience, and long‐term adjustment within university settings.

### 4.1 Practical Implications

We expect academic adjustment to significantly influence various aspects of students' educational experiences, fostering their psychosocial growth and overall success. A comprehensive exploration of the diverse factors that facilitate this adjustment is essential for understanding the concepts and potential benefits associated with academic adjustment (Kiang and Fuligni 2010). The findings of this study indicate that academic hope relates to academic adjustment in higher education. Academic hope, when mediated by academic engagement, significantly enhances students' adjustment to their educational environment.

Our results also reveal that male students exhibit higher levels of academic hope than their female counterparts, which correlates with improved academic adjustment in the educational context. We recommend that educational professionals develop and implement targeted strategies to enhance academic hope and adjustment, particularly among female students. Increasing engagement in educational activities fosters greater hope among all students, which in turn leads to improved adjustment within the academic environment. Understanding these dynamics enables students to navigate their academic journeys more successfully and harmoniously.

### 4.2 Limitation and Future Research

This study focused exclusively on undergraduate students at public universities in Tehran. Therefore, we should approach the generalization of the results to students at other academic levels or private universities with caution. The study did not consider additional demographic variables, such as field of study, socioeconomic status, and personal characteristics, which could influence the results. Given the correlational nature of this study, one should exercise caution when drawing causal inferences from the findings. As a cross‐sectional study, it cannot adequately demonstrate causal relationships between the variables.

A further limitation is the possibility of conceptual overlap between motivation and academic hope. Despite the theory‐based distinction between the constructs, the Academic Motivation Scale could have included items tapping hope‐related components, such as going for a goal or persistence. This overlap could have affected the observed associations. Future studies would be wise to use more refined measures or analytical approaches in order to disentangle the distinct contribution of each construct more effectively.

For future research, we recommend conducting similar studies across different academic levels and private universities to enhance the generalizability of the findings. Longitudinal studies aimed at examining causal relationships between variables and tracking changes over time could yield more valuable insights. Given the moderating role of gender in certain pathways, further investigations focusing on gender differences in these relationships may provide additional information. Addressing these limitations in future studies can offer recommendations for research in this field and, more broadly, explore the role of academic adjustment within various aspects of the educational environment.

## 5 Conclusion

The findings of our study enhance the understanding of strategies for improving academic adjustment for educators and students. This research investigates the mediating role of academic engagement in the relationship between academic hope and adjustment. To examine this mediating role, we developed a structural model and employed SEM for analysis. The results reveal that academic hope significantly explains variance in academic adjustment and is associated with academic adjustment among higher education students. The final model supports the mediating role of academic engagement in the relationship between academic hope and adjustment, suggesting that academic hope is linked to students' adjustment to the educational setting through engagement. Our findings indicate that gender moderates the effect of academic hope on academic adjustment, revealing significant differences between male and female students.

Thus, increasing students' academic hope can enhance academic engagement, ultimately leading to improved academic adjustment. These findings can inform the design of interventions, educational programs, and counseling strategies aimed at facilitating students' academic adjustment. Taking into account gender differences in this context is crucial for the development of these programs, which will help students navigate their academic journeys successfully.

## Author Contributions

In the current research the Maryam Mohsenpour, as a supervisor, supervised and strategized the overall process of the research, the Kimia Mehrali has been responsible for developing the research plan, collecting and sifting the data, writing the text of the article, drawing conclusions from the findings, expanding, and interpreting. The Abbas Abdollahi supervised the analysis and interpretation of the data. All authors read and approved the final manuscript.

## Funding

The authors have nothing to report.

## Conflicts of Interest

The authors declare no conflicts of interest.

## Ethics Approval and Consent to Participate

This article is based on the Master's thesis of the first author, completed under the supervision of the second author and the consultation of the third author. The study was conducted in accordance with the principles of the Declaration of Helsinki, informed consent was obtained from all participants prior to their inclusion in the study. It should be noted that the research proposal of the master thesis has been considered and received ethically approval for conducting by the faculty members of the educational psychology department at the faculty of education and psychology, Alzahra University on January 13, 2024. To ensure the privacy and anonymity of participants, no identifying information (such as names, student IDs, or class codes) was collected at any stage of data gathering. Participation in the study was entirely voluntary, and students were clearly informed that their responses would be used solely for research purposes and would remain completely anonymous. The questionnaires were completed privately in classroom settings and submitted in sealed envelopes to minimize any potential influence from instructors or peers.

## Data Availability

The data analyzed in this study that support these findings are available upon request from the corresponding author.
